# Genome-Wide Association Studies Revealed Several Candidate Genes of Meat Productivity in Saryarka Fat-Tailed Coarse-Wool Sheep Breed

**DOI:** 10.3390/genes15121549

**Published:** 2024-11-29

**Authors:** Kairat Dossybayev, Makpal Amandykova, Ainur Orakbayeva, Sholpan Adylkanova, Altynay Kozhakhmet, Kanagat Yergali, Temirlan Kulboldin, Beibit Kulataev, Aibyn Torekhanov

**Affiliations:** 1 LLP «Kazakh Research Institute of Animal Husbandry and Fodder Production», 51 Zhandosov Street, Almaty 050071, Kazakhstan; kairat1987_11@mail.ru (K.D.); altynaitg@gmail.com (A.K.); ergaly.qanagat@gmail.com (K.Y.); k.temoha@gmail.com (T.K.); bnar68@yandex.ru (B.K.); torehanov.aibyn@mail.ru (A.T.); 2 Faculty of Veterinary Medicine and Zooengineering, Kazakh National Agrarian Research University, 8 Abai Avenue, Almaty 050010, Kazakhstan; ainura_manat@mail.ru (A.O.); adylkanovasholpan@maiil.ru (S.A.); 3 RSE Institute of Genetics and Physiology SC MSHE RK, 93 Al-Farabi Avenue, Almaty 050060, Kazakhstan

**Keywords:** Kazakh fat-tailed sheep, sheep meat productivity, GWAS, candidate genes

## Abstract

Background: Saryarka sheep belong to fat-tailed coarse-wool sheep breed. This breed is distinguished by increased meat productivity while being competitive in young lamb production. Live weight and body indices are relevant data for assessing sheep body constitution, which directly affects the breeding characteristics and meat productivity of animals. Objectives: This study aimed to find associations with SNPs and nine phenotypic characteristics of the Saryarka fat-tailed coarse-wool sheep breed including live weight and eight body indices (wither height, rump height, bicoastal diameter, body depth, body length, rump width, heart girth, and cannon bone circumference), and find candidate genes related to these characteristics. Methods: A total of 100 animals from the Karaganda region of Kazakhstan were used in this study. Live weight and eight body indices of sheep were measured using tape and electronic scales. The blood samples of the animals were used for DNA extraction. DNA samples were genotyped with the OvineSNP50 Genotyping BeadChip and analyzed using GWAS. Statistically significant SNPs were identified for each characteristic trait referencing the genome of *Ovis aries* (Oar_v3.1) using BioMart. Results: The GWAS results demonstrated a substantial chromosomal-level correlation between 32 chromosome-wide significant and suggestively significant SNPs in the studied sheep breed. Overall, seven SNPs located in seven different genes were revealed as candidates for live weight and four body indices: s20793.1 SNP in the *IGFBP6* gene for live weight, OAR4_54217431.1 SNP in the *ST7* gene for bicoastal diameter, s25229.1 in the *SCD5* gene, and s01175.1 SNP in the *DTNBP1* gene for rump width, OAR2_175574781.1 SNP in the *KYNU* gene for heart girth, and OAR1_209022621.1 SNP in the *FGF12* gene and s15415.1 SNP in the *FTO* gene for cannon bone circumference. Some of these genes were previously reported to be involved in body constitution and fat deposit in other sheep breeds. Conclusions: The results of the present study open up new opportunities for targeted sheep breeding for meat and fat productivity.

## 1. Introduction

Sheep farming has been an integral part of the Kazakh people’s culture since ancient times and is currently one of the most important branches of livestock production in Kazakhstan. Saryarka fat-tailed sheep is a meat–fat breed widespread in Central and South-East parts of the country. This breed originated in 1999 as a cross of the Kazakh coarse-wool sheep breed ewes and the Edilbay sheep breed rams. The breed is 8–10% superior to local Kazakh coarse-wool sheep in meat and fat production while having the same wool productivity indices. The live weight of rams is 95–110 kg, and wool shearing is 2.8–3.2 kg; as for ewes, it is 60–65 kg and 2.0–2.4 kg, respectively [1] (p. 1).

Extensive genetic research contributes to the sustainable development of sheep farming by improving important productivity traits like meat, milk, and wool. At the same time, it is important to exclude the overselection of growth traits at the expense of other important traits that can lead to undesirable outcomes in livestock such as health-related problems or the welfare of animals. It is important to understand that genetic selection of animals involves extensive work that includes not only selection for productivity traits but also monitoring the health of animals to prevent the loss of such important qualities as disease resistance and immunity. Thus, obtaining data on as many traits and their genetic background as possible can help solve this problem.

The achievements of modern molecular biology have made genome-wide association studies (GWASs) an important method of revealing candidate genes for different quantitative traits in animal husbandry [2] (p. 1). The data required for marker-assisted and gene-based selection are provided by the identification of SNPs and genes associated with productive qualities. SNPs enable the identification of genetic loci associated with traits of interest. Studies have shown that SNP-based GWASs help uncover genetic variants linked to traits like litter size, weight gain, and fertility, facilitating marker-assisted selection to improve productivity [3]. GWASs in sheep have been successfully carried out for several productive traits crucial for sheep breeding, such as live weight [2,4] (pp. 2–14), prolificacy [5], body height and length, chest circumference [2] (pp. 2–14), wool, skin wrinkle and breech cover traits [6], and even blood parameters [7]. The OvineSNP50 Genotyping BeadChip has proven effective in genetic studies of sheep by enabling high-resolution GWASs and identifying significant SNP loci related to economically important traits such as growth and reproduction [8]. Several studies were performed using the OvineSNP50 for the population structure analysis of sheep [9], including on Kazakh sheep breeds [10,11]. These investigations extended insights into genetic diversity and differences in breeds. However, none of them included GWAS methods to search for candidate associations (SNPs, genes) for traits affecting sheep productivity.

Body morphological characteristics (live weight and body indices) are significant economic characteristics in sheep since they are linked to meat production and growth [12] (p. 561). The most significant growth and development index in sheep productivity is body weight, which has an impact on meat and wool productivity as well as sheep reproduction. A growth in body weight is influenced not only by feeding and management but also by genetic background. Thus, weight gain is one of the main indices of selection while being a highly heritable feature [13]. Body weight is considered the most important trait for meat productivity, especially in sheep [14]. At the same time, birth weight is the earliest available trait that directly impacts the ability of a lamb to survive [15]. Three genes (*RAB6B*, Tf serotransferrin, and *GIGYF2*) on chromosome 1 were revealed as the candidate genes for live weight in Lori-Bakhtiari sheep [4]. In addition, four genes (*AADACL3* (chromosome 12), *VGF* (chromosome 24)*, NPC1* (chromosome 23)*,* and *SERPINA12* (chromosome 18)) in Chinese Fine-Wool Sheep [16] and three genes (*CAPN6* (*chromosome* 27), *ITGA11* (*chromosome* 7), and *SCMH1* (*chromosome* 1)) in Hu sheep were associated with body weight in different age stages of sheep including birth, weaning, yearling, and adult weight [17].

Another important criterion for assessing the proportionality of the exterior at different age periods is the physique indices. Measuring the body indices allows us to estimate sheep growth, development, and changes in body proportions at different ages; compare the exterior characteristics of ancestors and their descendants; and make conclusions about differences in body type according to their correspondence to a certain productivity direction. Sheep breeds in different productivity directions have clearly defined exterior features that must be considered when evaluating productivity traits. For example, meat-fat sheep should have well-defined body shapes (deep and wide chest, wide and fairly long back and lower back, and correct positioning of the limbs) [18] (pp. 29–34). Animal live weight has often been estimated using body measures, particularly in situations where weighing equipment is not available. External body measures have been used in practice to estimate how the body’s soft tissues and/or skeleton have developed. For example, the prediction of the body weight of Nilotic sheep can be based on heart girth measurements [19].

Growth traits are quantitative traits influenced by numerous genes and assessed by different phenotypic traits like live weight, wither height, and body length. There are no GWAS data on the Saryarka fat-tailed sheep breed of Kazakhstan. Thus, our research goal was to identify candidate genes associated with live weight and eight body indices in Saryarka fat-tailed sheep using a GWAS.

## 2. Materials and Methods

### 2.1. Experimental Animals and Phenotypic Data Collection

A total of 100 animals of 18-month-old Saryarka fat-tailed sheep from the Zhenis collective farm located in the Ulytau district of the Karaganda region of Kazakhstan were selected for this study. Live weight (LW) and eight body indices (wither height (WH), rump height (RH), bicoastal diameter (BCD), body depth (BD), body length (BL), rump width (RW), heart girth (HG), and cannon bone circumference (CBG)) of the animals were measured using tape measures and electronic scales. The description of each index [18] (pp. 29–34) is shown in Table 1.

A PCA plot [20] was built to present the clusters of the morphometric traits.

Blood samples for DNA extraction were collected by veterinarians in 9 mL EDTA vacuum tubes (Leuven, Belgium) to prevent blood coagulation and directly transported to the laboratory of molecular-genetic testing of the LLP «Kazakh Research Institute of Animal Husbandry and Fodder Production» in special thermally insulated boxes with a refrigerant. All ethical standards prescribed for animal research were observed during biomaterial collection per the approval of the Local Bioethics Committee of the LLP «Kazakh Research Institute of Animal Husbandry and Fodder Production» (Conclusion of the Bioethics Committee №11 from 5 April 2022, Almaty, Kazakhstan).

### 2.2. DNA Extraction and Quality Control

DNA isolation was performed using the GeneJET Genomic DNA Purification Kit (Thermo Scientific, Waltham, MA, USA) according to the manufacturer’s protocol. Quality control of the extracted DNA was implemented on a NanoDrop One spectrophotometer and Qubit Fluorometer (Thermo Scientific, USA). DNA concentration was quantified to 50–100 ng/L for SNP genotyping.

### 2.3. SNP Genotyping and Quality Control

SNP genotyping was performed using the OvineSNP50 Genotyping BeadChip with a coverage of 54,241 SNPs (Illumina, San Diego, CA, USA) on the iScan system (Illumina, USA). Map and ped format files were generated using OvineSNP50 genotyping data and were subjected to data quality control on PLINK 1.90 [21] (p. 1). Several quality control criteria recommended for GWAS were used [22] to exclude low-quality SNPs: missingness of SNPs and individuals (--geno 0.2, --mind 0.2, --geno 0.02, --mind 0.02), SNPs under the set minor allele frequency threshold (--maf 0.05), markers that deviate from Hardy–Weinberg equilibrium (--hwe 1e-6), and the pruning of a subset of markers that are in approximate linkage equilibrium with each other (--indep-pairwise 50 5 0.2).

### 2.4. Genome-Wide Association Studies and Candidate Gene Annotation

The GWAS was performed on PLINK 1.90 [21] (p. 1), fully accounting for individual relationships and population stratification. Statistically significant SNPs were identified for each characteristic trait referencing the genome of *Ovis aries* (Oar_v3.1) using BioMart [23]. A Manhattan plot was built using R Package v.4.4.1 [24]. Bonferroni correction was used to control the family-wise error rate [25]. The suggestive association significance threshold was *p* < 0.05 (*p* < 5 × 10^−8^) at the chromosome-wide level. The significant association indicates that the chromosome-wide level association considered corresponds to a p-value less than 10^−5^. The p-value indicator for each significant and suggestively significant SNP is presented in the Results part of this paper. The quantile–quantile (Q–Q) graphics were plotted using the distribution of obtained and expected log10 (*p*-value) with inflation factors (λ). Q–Q plots were also plotted with R Package v.4.4.1.

## 3. Results

### 3.1. Phenotypic Data

Live weight and eight body size traits were measured from 100 Saryarka sheep. The live weight indicators ranged from 42.8 to 58.1 kg, with an average of 50.6 kg in the studied individuals. The calculated results showed that the wither and rump height values were similar. The mean values of BCD, BD, BL, and RW were 21.95, 30.69, 82.96, and 22.51 cm, respectively. The heart girth was between 86.0 and 102.0 cm, and the CBG was between 8.0 and 11.0 cm (Table 2).

**Figure 1:**
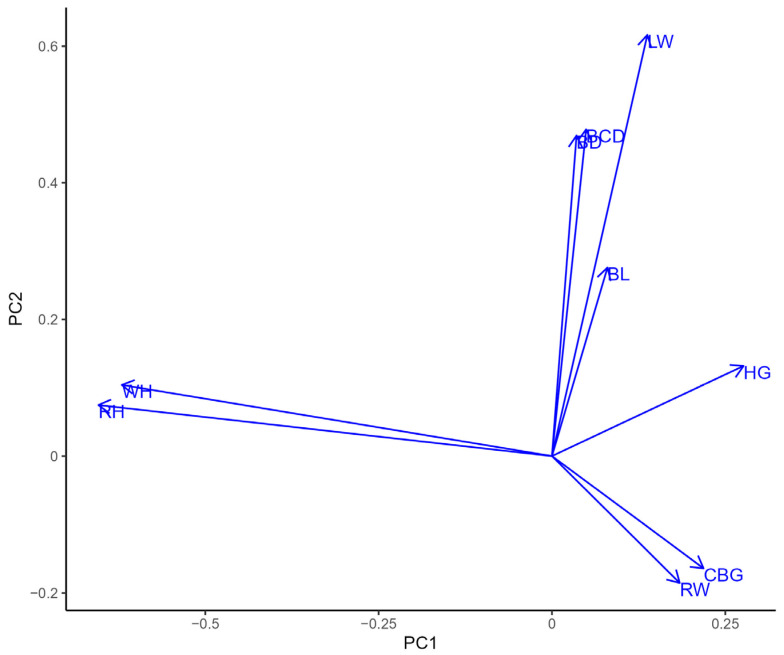
Principal component analysis of the physical characteristics of the studied sheep.

The PCA plot presents the morphometric traits clustered into three clusters (Figure 1).

A high association was found between CBG and RW, while the second significant relationship was between RH and WH, and all remaining indices clustered together.

### 3.2. SNP Quality Control Results

As a result of SNP genotyping of 100 animals, 51,135 SNPs were obtained and subjected to several steps of quality control. After excluding SNPs and individuals with low genotype calls in two steps (--geno 0.2 and --mind 0.2 and --geno 0.02 and --mind 0.02), 49,719 variants were selected for further QC steps. Exclusion of SNPs under the set MAF threshold (--maf 0.05) resulted in 44,472 variants, and after removing the markers deviating from the Hardy–Weinberg equilibrium (--hwe 1.0 × 10^-6^), 98 sheep and 44,470 SNPs were passed for the final QC step of pruning (--indep-pairwise 50 5 0.2). As a result, 95 sheep and 34,463 SNPs were referred for the final analysis. These results show a high quality of most of the obtained SNPs.

### 3.3. Genome-Wide Association Analysis and Gene Annotation Results

**Figure 2:**
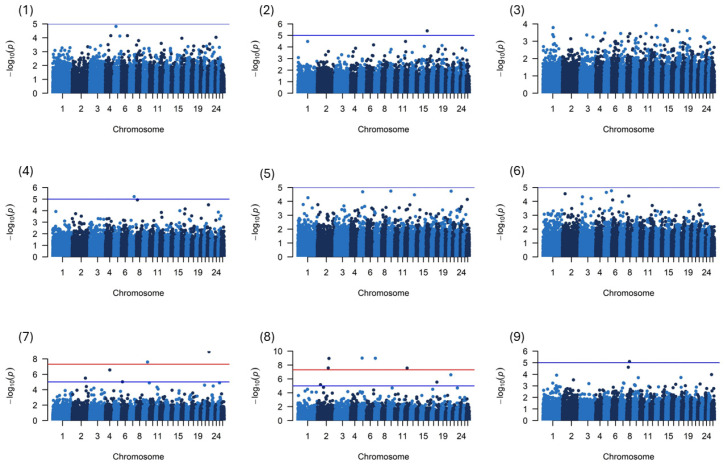
Manhattan plots of GWASs of nine physical characteristics of Saryarka fat-tailed sheep: (**1**) LW; (**2**) WH; (**3**) RH; (**4**) BCD; (**5**) BD; (**6**) BL; (**7**) RW; (**8**) HG; (**9**) CBG. The horizontal red line represents the genome-wide significance level threshold, and SNPs located above the red line indicate reaching the genome-wide significance level.

Figure 2 shows the Manhattan plot of nine physical characteristics of Saryarka fat-tailed sheep with 34,463 SNPs distributed on 26 chromosomes (the significance level threshold after correction was 1.0 × 10^−5^). The quantile–quantile plots which characterize the distribution of the observed *p*-values (−log10 *p*-values) of SNPs against the expected values are shown in Supplementary Figure S1.

The observed values of the studied traits deviated from the expected values with different inflation factor indicators (λ). This indicator was approximately similar for all studied physical characteristics, ranging from 0.89332 to 1.20852.

The GWAS results demonstrated a substantial chromosomal-level correlation between 32 chromosome-wide significant and suggestively significant SNPs. Seven SNPs were located within the genes including *IGFBP6*, *ST7*, *DTNBP1*, *SCD5*, *KYNU*, *FGF12*, and *FTO* (Supplementary Figure S2). Seventeen SNPs were not positionally linked to a gene and no matches with the reference genome were found for the remaining eight SNPs, although they had a significant association level (Table 3).

No significant and suggestive SNPs were found for the wither height of Saryarka fat-tailed sheep. Therefore, the research results and discussion will only be presented for the eight phenotypic features.

#### 3.3.1. Live Weight

Four suggestively associated SNPs were found for the Saryarka fat-tailed sheep’s live weight. Three SNPs (s38034.1, OAR6_96649415.1, and OAR5_95756483.1) were not positionally linked to a gene, while s20793.1 SNP was located within the *IGFBP6* (insulin-like growth factor-binding protein 6) gene in chromosome 3 (*p* = 0.001312).

#### 3.3.2. Rump Height

Only two chromosome-wide suggestively associated SNPs were detected for RH on chromosomes 1 and 17. However, no genes were annotated for them.

#### 3.3.3. Bicoastal Diameter

No SNPs surpassed the chromosome-wide significance threshold for associations with BCD. However, two SNPs located on chromosomes 4 and 15 were suggestively associated with this body index. OAR4_54217431.1 SNP on chromosome 4 was positioned within the *ST7* (suppression of tumorigenicity 7) gene, while s59091.1 SNP on chromosome 15 was not positionally linked to a gene.

#### 3.3.4. Body Depth

Only one suggestively associated SNP (s55235.1) was found for BD in Saryarka fat-tailed sheep. Nevertheless, it was not located within the gene.

#### 3.3.5. Body Length

This study revealed three SNPs suggestively associated with BL in Saryarka fat-tailed sheep: OAR5_114683952_X.1 (chromosome 5), OAR2_40125657.1 (chromosome 2), and s06635.1 (chromosome 3).

#### 3.3.6. Rump Width

Five significant and two suggestively significant SNPs were detected for RW in the studied sheep breed. All significant SNPs (OAR2_199679972.1, OAR4_65928409.1, OAR6_20647551.1, OAR9_71374559.1, and s39884.1) located on chromosomes 2, 4, 6, 9, and 22, respectively, were not positionally linked to a gene. Furthermore, we identified two suggestively correlated SNPs for rump width, located within the *SCD5* (Stearoyl-CoA Desaturase 5) and *DTNBP1* (dystrobrevin binding protein 1) genes on chromosomes 6 and 20, respectively.

#### 3.3.7. Heart Girth

HG presented the highest number of associated SNPs with eight significant SNPs located on chromosomes 2, 5, 7, 12, 18, and 21. OAR2_175574781.1 SNP was located within the *KYNU* (kynureninase) gene of chromosome 2 at position 165981432 bp (A<G mutation). Four SNPs (s55928.1, OAR2_165244995.1, OAR12_37292970.1, and OAR21_33775912_X.1) were not mapped in genes and three SNPs (OAR7_11868747.1, OAR18_11476519.1, and OAR2_48251746.1) did not have any matches with the reference genome in gene ontology.

#### 3.3.8. Cannon Bone Circumference

One significantly and four suggestively associated SNPs were found for CBG in Saryarka sheep. The OAR8_42165866.1, OAR8_25841996.1 (both in chromosome 8), and s49020.1 (chromosome 17) SNPs were not annotated within the genes. However, the results showed an association between the OAR1_209022621.1 SNP in the *FGF12* (Fibroblast Growth Factor 12) gene (chromosome 1) and s15415.1 SNP in the *FTO* (fat mass and obesity) gene (chromosome 14) and the CBG.

## 4. Discussion

The Saryarka sheep breed is widely used to increase meat productivity and early maturity and to lighten the wool color in meat-and-fat coarse-wool sheep breeding in Kazakhstan [26]. The body parameters can be used as a reliable trait for predicting the body weight and body size of sheep. In turn, body size and tail fat are the main indicators of the meat productivity of sheep [12] (p. 561). Thus, expanding knowledge of the genetic characteristics of the breed, especially important traits for productive sheep breeding, is a prerequisite for the intensive development of livestock production. To our knowledge, this is the first study performing large-scale GWASs in Saryarka sheep using the OvineSNP50 genotyping BeadChip for several phenotypic indices determining the productivity of animals.

In this study, we used 100 animals of the Saryarka fat-tailed sheep breed for GWASs to find candidate genes and SNP associations with live weight and eight body indices that directly affect sheep meat productivity. Overall, we identified seven SNPs located in the *IGFBP6*, *ST7*, *DTNBP1*, *SCD5*, *KYNU*, *FGF12*, and *FTO* genes that were suggestively associated with five specific body indices in sheep: LW, BCD, RW, HG, and CBG.

### 4.1. Live Weight

The GWASs showed a high association between SNP s20793.1 and live weight in Saryarka fat-tailed sheep. This SNP was located in the *IGFBP6* gene, which was previously speculated to be a cross-species tendon marker in mammals [27]. Interestingly, the tendon is well developed in Kazakh fat-tail sheep breeds as these sheep graze year-round outdoors and use about 15–20 km daily for forage supply. Thus, a more detailed study of the identified SNP may be a precursor to its use in evaluating sheep tendons to improve the long-distance walking ability of animals in grazing systems. Moreover, it was reported that the *IGFBP2* and *IGFBP5* genes, which are part of the same gene family as *IGFBP6*, are both involved in regulating IGF action, which influences the rate of growth and development [28]. This suggests that IGFBP6 may be a strong candidate for the selection of sheep for meat production genes. Other previous studies on the search for associations between animals’ weight at different stages of development also reported several genes associated with this trait including the *OAR2* gene in Akkaraman fat-tailed sheep [29]; *FAM184B*, *NCAPG*, and six other genes in Alpine Merino Sheep [30]; and the *TNC*, *TNFSF8*, and *COL28A1* genes in Romney sheep [31]. Different environmental and physiological conditions of animals, as well as the different genetic backgrounds of the sheep breeds in these studies, might affect the differences in the results.

### 4.2. Bicoastal Diameter

Bicoastal diameter plays an important role in the assessment of muscle tissue development, which directly affects the meat productivity of sheep. Earlier studies reported several candidate genes related to this indicator [17,32] (p. 588). However, the *ST7* gene found to be a candidate gene for BCD in the present study is a candidate gene newly observed in sheep. Its function has not been determined yet in mammals [33]. Therefore, it might be interesting to explore it deeper and explain its relatedness with sheep bicoastal diameter in future works.

### 4.3. Rump Width and Cannon Bone Circumference

Furthermore, our study revealed an association between the s25229.1 SNP (located within the *SCD5* gene) and the rump width index in Saryarka sheep. The rump width is one of the most relevant body parameter traits for fat-tailed sheep. The *SCD5* gene plays a central role in sheep body size development [34,35] (pp. 121–127, pp. 1–6) and is linked to the regulation of sterol-regulatory element-binding proteins involved in body size development [34] (pp. 121–127). Interestingly, its association with body size was also revealed in Kazakh Edilbai and Shetland sheep breeds [35] (pp. 1–6). Considering that the body constitution of animals directly influences the amount of meat obtained at slaughter, this SNP can be considered a strong candidate for sheep body size determination.

The *DTNBP1* and *FTO* genes were reported to be involved in fat metabolism and tail deposition in sheep [36,37] (p. 361, p. 2). The *DTNBP1* gene has also been associated with fat metabolism in the Hulun Buir sheep breed [36] (pp. 361–367), while the *FTO* gene was revealed to be involved in tail fat deposition in Hu sheep [37] (pp. 1–6). Fat reservation in the organism during favorable forage conditions of the year is the most valuable adaptive feature of meat-and-fat sheep for breeding in the desert and semi-desert areas. Fat deposits are created mainly at the root of the tail in the form of a pillow and are called tail fat. Often, the weight of the tail fat reaches 15–20 kg or more in Saryarka fat-tailed sheep. The tail fat is consumed by sheep during starvation when the grass is burnt out or covered with snow, as well as in summer during interruptions in water supply. In terms of taste and nutritional qualities, tail fat is unlike internal and subcutaneous fat [1] (p. 1). Consequently, a more detailed study of the role of SNPs found in the *DTNBP1* and *FTO* genes may open up opportunities for breeding programs aimed at fat-tailed sheep breeding.

The OAR1_209022621.1 SNP in the *FGF12* gene was associated with the CBC of Saryarka sheep. To date, there are no studies to reveal the role of the *FGF12* gene in sheep body constitution. However, the *FGF12* gene was earlier described as a candidate for fiber length in Spanish Merino sheep [38] (pp. 1–10), and in another study, this gene was suggested to have an essential role in the hair growth cycle correlating with hair length in humans. In addition, the *FGF12* gene was associated with the staple length of Spanish Merino sheep [38] (pp. 1–10). Saryarka is a coarse-wool sheep breed; its fast wool growth makes it possible to shear the animals twice a year. Therefore, further study of the effect of the *FGF12* gene on wool might be interesting for Saryarka sheep.

### 4.4. Heart Girth

The *KYNU* gene encoding an enzyme within the tryptophan metabolism pathway [39], which influences animal growth, was associated with HG in Saryarka fat-tailed sheep. A previous study demonstrated that tryptophan was the third most limiting amino acid in growing lambs, and its inadequate supply or increased catabolism in vivo can trigger limited protein deposition and the elevation of urinary N excretion [40]. The altered abundance of kynureninase involved in protein metabolic function, integrated with the changes in serum levels of blood urea nitrogen and glucose, suggests that overgrazing triggers a shift in energy resources from carbohydrates to proteins, causing poorer nitrogen utilization efficiency. Altogether, it suggests that the reductions in animal growth induced by overgrazing are associated with liver proteomic changes, especially the proteins involved in nitrogen compound metabolism and immunity [41]. Thus, we may assume that the OAR2_175574781.1 SNP within the *KYNU* gene identified in our study can be used as a strong candidate to reveal not only the growth development but also the welfare of the lambs.

The results of our research provide new data on candidate SNPs for most sheep body constitution indices that directly determine the meat, fat, and wool productivity of sheep. Our study results detected new SNPs and candidate genes different from those found in previous studies. This might be explained by the study’s inclusion of only one sheep breed and the fact that candidates for the studied phenotypic features may vary among different breeds. The number of the studied animals may also be one of the factors of limitations that may affect the results of the study. Although current findings need to be validated extensively by increasing the sample number of the studied animals or including other sheep breeds to compare findings, they promote our understanding of the association between zootechnical characteristics and underlying genetic mechanisms. Moreover, they allow us to use this knowledge in the sustainable development of meat productivity in sheep husbandry.

## 5. Conclusions

Our study illustrates seven chromosome-wide significant SNPs associated with five different body indices (live weight, bicoastal diameter, rump width, heart girth, and cannon bone circumference) in Saryarka fat-tailed sheep. These SNPs were located in seven genes, namely *IGFBP6*, *ST7*, *DTNBP1*, *SCD5*, *KYNU*, *FGF12*, and *FTO*. The study results establish an important foundation for molecular-assisted breeding targeting live weight and body indices in sheep and can be widely used as selection criteria in sheep breeding. Considering that the live weight and body indices are directly related to the meat productivity of sheep, the resultant breeding approaches may produce more productive animals. To gain more sophisticated knowledge of the genetic mechanisms of body indices and to perform a functional verification of identified genes, it is recommended to conduct research in other sheep populations.

## Figures and Tables

**Table 1:** Description of studied physical characteristics of Saryarka fat-tailed sheep.

№	Physical Characteristics	Description
1	Live weight	The weight of an animal after completion of rearing and feeding
2	Wither height	Determined by the measurement of the highest point of the withers in a straight line from the ground
3	Rump height	Determined by the measurement of the highest point of the sacrum
4	Bicoastal diameter	Determined by the measurement of the widest point vertically, tangent to the rear angle of the scapula
5	Body depth	Determined by the measurement of the vertical distance from the highest point of the withers behind the shoulder blades to the lower surface of the chest
6	Body length	Determined by the measurement of the distance from the extreme anterior point of the scapulohumeral joint to the inner protrusion of the ischial tuberosity
7	Rump width	Measured as the distance between the most posterior point of the pin bones
8	Heart girth	Determined by the measurement around the circumference, passing tangent to the back angle of the shoulder blade
9	Cannon bone circumference	Determined by the measurement of the girth at the thinnest point of the metacarpal bone

**Table 2:** Descriptive statistics of the physical characteristics of the Saryarka fat-tailed sheep breed.

№	Physical Characteristics	Sample Size	Mean	Min. Value	Max. Value
1	LW, kg	100	50.601 ± 3.924	42.8	58.1
2	WH, cm	100	71.205 ± 1.432	67.0	73.8
3	RH, cm	100	71.816 ± 1.778	65.0	74.2
4	BCD, cm	100	21.95 ± 1.579	18.0	24.0
5	BD, cm	100	30.69 ± 1.846	25.0	35.0
6	BL, cm	100	82.96 ± 3.843	72.0	90.0
7	RW, cm	100	22.55 ± 2.143	19.0	27.0
8	HG, cm	100	94.57 ± 4.115	86.0	102.0
9	CBG, cm	100	9.354 ± 0.893	8.0	11.0

**Table 3:** Genome-wide association study results and candidate genes of the phenotypic traits of the Saryarka fat-tailed sheep breed.

№	Phenotypic Characteristics	Chr	SNP in Gene	Candidate Genes	Related SNPs	Position, bp	Substitution	*p*-Value	Association Level
1	LW	4	No	*-*	s38034.1	76916480	T<C	0.00007048	Suggestive
		6	No	-	OAR6_96649415.1	88113526	A<G	0.00007065	Suggestive
		5	No	-	OAR5_95756483.1	87568490	G<A	0.00007584	Suggestive
		3	Yes	*IGFBP6*	s20793.1	133214823	C<T	0.001312	Suggestive
2	WH	No significant and suggestive SNPs were found							
3	RH	1	No	-	OAR1_155107985.1	143433100	T<C	0.0001638	Suggestive
		17	No	-	OAR17_39581977.1	36612240	C<T	0.0002826	Suggestive
4	BCD	15	No	*-*	s59091.1	64420672	G<T	0.0001018	Suggestive
		4	Yes	*ST7*	OAR4_54217431.1	51203290	C<T	0.0004799	Suggestive
5	BD	9	No	-	s55235.1	42238463	G<A	0.000018	Suggestive
6	BL	5	No	-	OAR5_114683952_X.1	105265869	C<A	0.0000171	Suggestive
		2	No	-	OAR2_40125657.1	38578195	T<C	0.00002798	Suggestive
		3	No	-	s06635.1	37310135	C<T	0.00004734	Suggestive
7	RW	22	-	-	s39884.1	27144608	A<G	0.00000000115	Significant
		9	-	-	OAR9_71374559.1	67241520	G<A	0.0000000254	Significant
		4	-	-	OAR4_65928409.1	62315843	G<A	0.000000277	Significant
		2	-	-	OAR2_199679972.1	188291939	A<G	0.00000318	Significant
		6	No	-	OAR6_20647551.1	17680070	G<A	0.00000952	Significant
		6	Yes	*SCD5*	s25229.1	97139005	A<G	0.0004653	Suggestive
		20	Yes	*DTNBP1*	s01175.1	40381688	T<C	0.002427	Suggestive
8	HG	5	No	-	s55928.1	45763719	C<T	0.000000000993	Significant
		7	-	-	OAR7_11868747.1	11579221	A<G	0.00000000106	Significant
		2	Yes	*KYNU*	OAR2_175574781.1	165981432	A<G	0.0000000011	Significant
		2	No	-	OAR2_165244995.1	155873954	A<G	0.0000000266	Significant
		12	No	-	OAR12_37292970.1	33220261	A<G	0.0000000277	Significant
		21	No	-	OAR21_33775912_X.1	30148681	G<T	0.000000256	Significant
		18	-	-	OAR18_11476519.1	11480063	G<A	0.00000288	Significant
		2	-	-	OAR2_48251746.1	45537012	C<T	0.00000678	Significant
9	CBG	8	-	-	OAR8_42165866.1	39156805	C<T	0.000007901	Significant
		8	No	*-*	OAR8_25841996.1	23361179	T<G	0.00002434	Suggestive
		1	Yes	*FGF12*	OAR1_209022621.1	193436737	T<C	0.0001194	Suggestive
		17	No	-	s49020.1	65960779	A<C	0.0001891	Suggestive
		14	Yes	*FTO*	s15415.1	21730916	A<C	0.003363	Suggestive

## Data Availability

The original contributions presented in this study are included in the article/Supplementary Material. Further inquiries can be directed to the corresponding author(s).

## Supplementary Materials

The following supporting information can be downloaded at https://www.mdpi.com/article/10.3390/genes15121549/s1: Figure S1: The quantile–quantile plots of the GWASs of nine physical characteristics of Saryarka fat-tailed sheep. Figure S2: Genetic location map of the candidate genes for meat productivity in Saryarka fat-tailed sheep.
