# Supplementary material for: Genome-Wide Association Studies Revealed Several Candidate Genes of Meat Productivity in Saryarka Fat-Tailed Coarse-Wool Sheep Breed
Source: Genes (Basel). 2024 Nov 29;15(12):1549. doi: 10.3390/genes15121549 (PMC11728008; doi:10.3390/genes15121549)
Supplement: Supplementary file 1 [file genes-15-01549-s001.zip › Supplementary Figure S1.pdf]

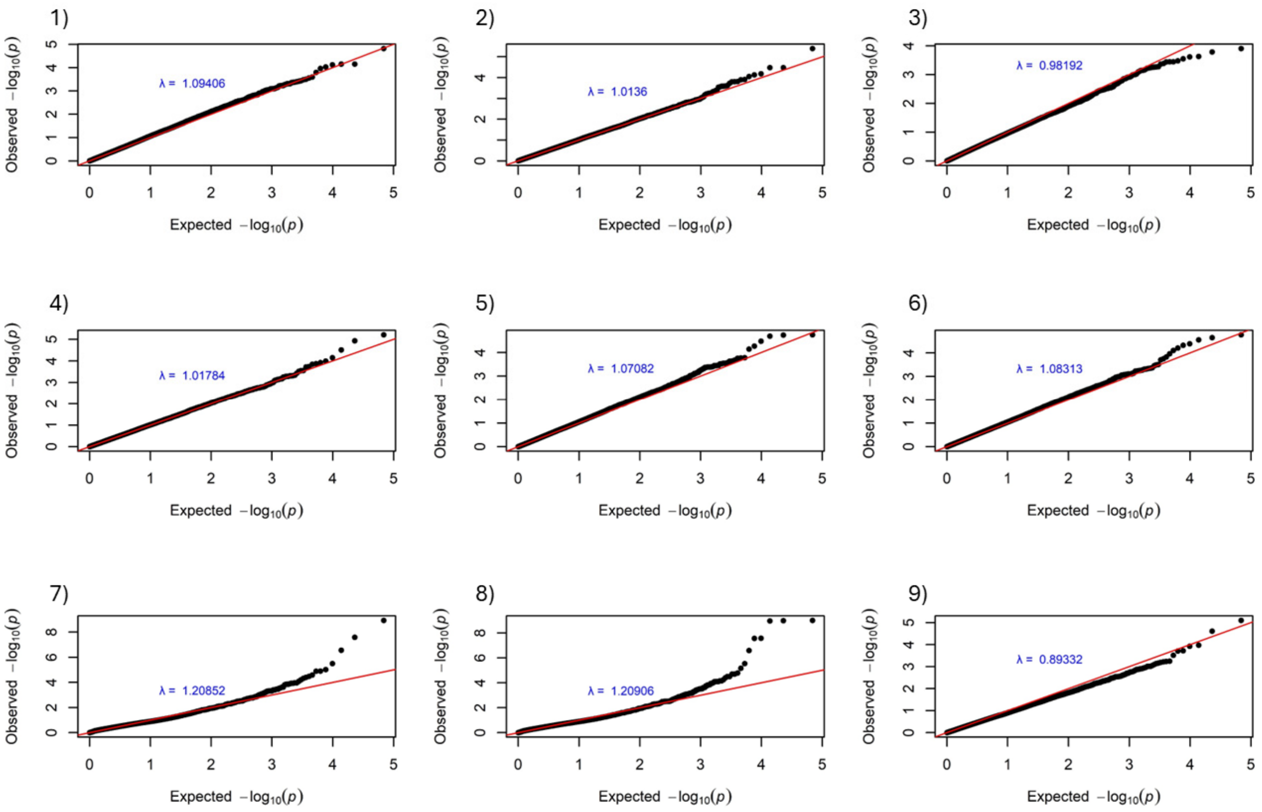

Supplementary Figure S1. The quantile-quantile plots of the GWAS of nine physical characteristics of Saryarka fat-tailed sheep: (1) LW; (2) WH; (3) RH; (4) BCD; (5) BD; (6) BL; (7) RW; (8) HG; (9) CBG. The red line in the quantile-quantile plots represents the genome-wide significance level threshold and blue line represents the suggestive line.
