# Supplementary material for: Genome-Wide Association Studies Revealed Several Candidate Genes of Meat Productivity in Saryarka Fat-Tailed Coarse-Wool Sheep Breed
Source: Genes (Basel). 2024 Nov 29;15(12):1549. doi: 10.3390/genes15121549 (PMC11728008; doi:10.3390/genes15121549)
Supplement: Supplementary file 1 [file genes-15-01549-s001.zip › Supplementary Figure S2.pdf]

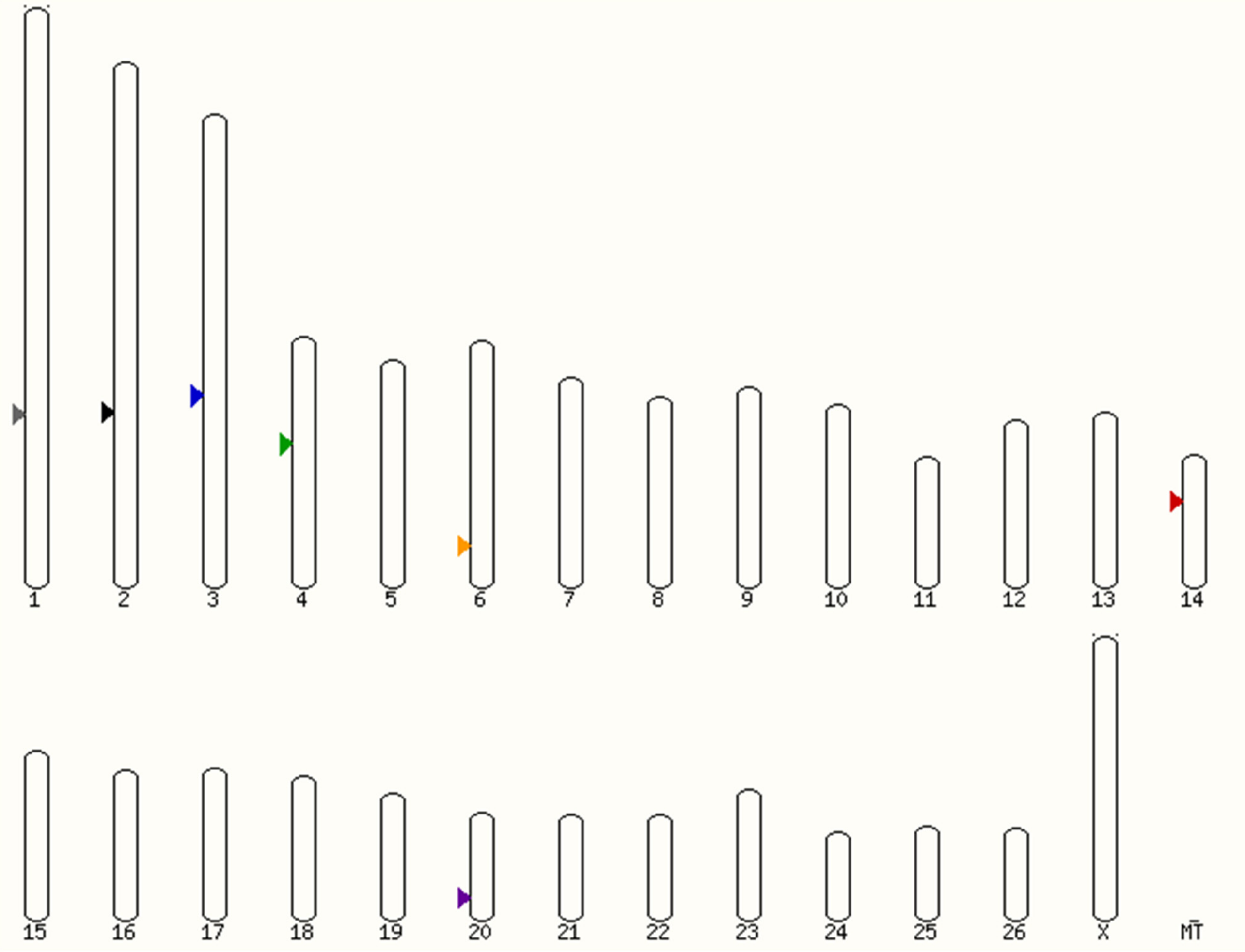

Supplementary Figure S2. Genetic location map of the candidate genes for meat productivity in Saryarka fat-tailed sheep: grey (1 chr) – *FGF12*; black (2 chr) – *KYNU*; blue (3 chr) – *IGFBP6*; green (4 chr) – *ST7*; orange (6 chr) – *SCD5*; red (14 chr) – *FTO*; purple (20chr) - *DTNBP1*
[https://asia.ensembl.org/Ovis_aries_texel/Location/Genome?time=1731781582]
